# Evaluating ChatGPT-4 for the Interpretation of Images from Several Diagnostic Techniques in Gastroenterology

**DOI:** 10.3390/jcm14020572

**Published:** 2025-01-17

**Authors:** Miguel Mascarenhas Saraiva, Tiago Ribeiro, Belén Agudo, João Afonso, Francisco Mendes, Miguel Martins, Pedro Cardoso, Joana Mota, Maria Joao Almeida, António Costa, Mariano Gonzalez Haba Ruiz, Jessica Widmer, Eduardo Moura, Ahsan Javed, Thiago Manzione, Sidney Nadal, Luis F. Barroso, Vincent de Parades, João Ferreira, Guilherme Macedo

**Affiliations:** 1 Precision Medicine Unit, Department of Gastroenterology, São João University Hospital, Alameda Professor Hernâni Monteiro, 4200-427 Porto, Portugal; tiagofcribeiro@outlook.com (T.R.); joaoafonso28@gmail.com (J.A.); francisco.cnm@gmail.com (F.M.); miguelpedro96@gmail.com (M.M.); pedromarilio@gmail.com (P.C.); joanamfmota8@gmail.com (J.M.); maj.almeida.14@gmail.com (M.J.A.); guilhermemacedo59@gmail.com (G.M.); 2 WGO Gastroenterology and Hepatology Training Center, Alameda Professor Hernâni Monteiro, 4200-427 Porto, Portugal; 3 Faculty of Medicine, University of Porto, Alameda Professor Hernâni Monteiro, 4200-427 Porto, Portugal; 4 Department of Gastroenterology, Hospital Universitario Puerta de Hierro Majadahonda, C/Joaquín Rodrigo, 28220 Madrid, Spain; bagucast@gmail.com (B.A.); antoniompintodacosta@gmail.com (A.C.); marianoghr@gmail.com (M.G.H.R.); 5 Division of Gastroenterology, NYU Langone Hospital—Long Island, 259 First Street Mineola, New York, NY 11501, USA; jessica.widmer@nyulangone.org; 6 Department of Gastrointestinal Endoscopy, Hospital das Clínicas da Faculdade de Medicina da Universidade de São Paulo, Rua Dr. Ovídio Pires de Campos 225, Sao Paulo 05403-010, Brazil; eduardoghdemoura@gmail.com; 7 Department of Colorectal Surgery, Royal Liverpool University Hospital, Liverpool L7 8YE, UK; ahsanj@liverpool.ac.uk; 8 Department of Surgery, Instituto de Infectologia Emílio Ribas, São Paulo 01246-900, Brazil; thiagomanzione@hotmail.com (T.M.); srnadal@terra.com.br (S.N.); 9 Internal Medicine/Infectious Diseases, Wake Forest University Health Sciences, Winston-Salem, NC 27109, USA; lbarroso@wakehealth.edu; 10 Department of Proctology, Hôpital Paris Saint-Joseph, 85, Rue Raymond Losserand, 75014 Paris, France; vdeparades@ghpsj.fr; 11 Department of Mechanical Engineering, Faculty of Engineering of the University of Porto, Rua Dr. Roberto Frias, 4200-465 Porto, Portugal; j.ferreira@fe.up.pt

**Keywords:** artificial intelligence, ChatGPT-4, convolutional neural networks, capsule endoscopy, device-assisted enteroscopy, endoscopic ultrasound, digital single-operator cholangioscopy, high-resolution anoscopy

## Abstract

**Background:** Several artificial intelligence systems based on large language models (LLMs) have been commercially developed, with recent interest in integrating them for clinical questions. Recent versions now include image analysis capacity, but their performance in gastroenterology remains untested. This study assesses ChatGPT-4’s performance in interpreting gastroenterology images. **Methods:** A total of 740 images from five procedures—capsule endoscopy (CE), device-assisted enteroscopy (DAE), endoscopic ultrasound (EUS), digital single-operator cholangioscopy (DSOC), and high-resolution anoscopy (HRA)—were included and analyzed by ChatGPT-4 using a predefined prompt for each. ChatGPT-4 predictions were compared to gold standard diagnoses. Statistical analyses included accuracy, sensitivity, specificity, positive predictive value (PPV), negative predictive value (NPV), and area under the curve (AUC). **Results:** For CE, ChatGPT-4 demonstrated accuracies ranging from 50.0% to 90.0%, with AUCs of 0.50–0.90. For DAE, the model demonstrated an accuracy of 67.0% (AUC 0.670). For EUS, the system showed AUCs of 0.488 and 0.550 for the differentiation between pancreatic cystic and solid lesions, respectively. The LLM differentiated benign from malignant biliary strictures with an AUC of 0.550. For HRA, ChatGPT-4 showed an overall accuracy between 47.5% and 67.5%. **Conclusions:** ChatGPT-4 demonstrated suboptimal diagnostic accuracies for image interpretation across several gastroenterology techniques, highlighting the need for continuous improvement before clinical adoption.

## 1. Introduction

The healthcare sector is being increasingly impacted by the introduction of commercially available artificial intelligence (AI) systems. Several computer-assisted detection or diagnosis systems (CADe or CADx, respectively) have been developed for medical fields, mainly when image interpretation is central to routine clinical practice. These systems are expected to expand healthcare access, improve outcomes, and provide a better experience to patients while lowering health-related costs.

Generative artificial intelligence (GAI) has been the focus of intense research in recent years. This interest has been motivated by the recent introduction of user-friendly large language models (LLMs), most notably Chat Generative Pre-Trained Transformer (ChatGPT, OpenAI). These AI models enable the interpretation of extensive textual data, and evidence confirms their potential in answering clinical questions, assisting differential diagnostic processes, providing patient recommendations, and elaborating research questions [1,2,3]. The introduction of ChatGPT-4 enabled new features, including image interpretation, combining textual and imaging data, thus providing multimodal predictions [4]. In fact, LLMs could be a potential solution for integrating both text and image analysis in a multimodal AI-based analysis, constituting a support decision system based on both patient data and images [5,6]. Nevertheless, despite their potential to generate clinical diagnoses when evaluating conventional textual data, integrating imaging data has provided suboptimal results.

The impact of LLMs on gastroenterology has only recently begun to be investigated. Indeed, most evidence focuses on evaluating the potential of these algorithms in solving clinical questions. In this setting, these algorithms have been shown to provide accurate outputs in delivering patient information about colonoscopy [7]. Moreover, ChatGPT provided adequate recommendations for gastroesophageal reflux disease in 91% of inputted questions [8].

The importance of AI in gastroenterology is more evident for gastrointestinal endoscopy. Indeed, AI algorithms based on convolutional neural networks (CNNs) have shown to be highly accurate in providing lesion detection and characterization during endoscopy exams [9,10]. The introduction of readily available LLMs with image interpretation functionalities is expected to improve lesion detection and facilitate clinical decisions. Nevertheless, to this date, the performance of LLMs for interpreting images in the field of gastroenterology remains to be evaluated. Thus, this study aimed to assess the performance of a widely available LLM (ChatGPT-4, OpenAI, San Francisco, CA, USA) the for automatic interpretation of images retrieved from gastroenterology procedure images, including capsule endoscopy, device-assisted enteroscopy, endoscopic ultrasound, digital cholangioscopy, and high-resolution anoscopy.

## 2. Methods

### 2.1. Study Design

A proof-of-concept study was designed to assess the performance of ChatGPT-4 (OpenAI) in the characterization of images retrieved from different gastroenterology procedures: capsule endoscopy, device-assisted enteroscopy, endoscopic ultrasound, direct single-operator cholangioscopy, and high-resolution anoscopy. This study included images from each diagnostic technique, which were submitted in batches for assessment by the LLM (Figure S1). Each image had a unique file name composed of a random number sequence to avoid potential bias in the LLM judgment. The use of endoscopic images was approved by local institutional review boards at each included institution (São João University Hospital [SJUH], Hospital Universitario Puerta de Hierro [HUPH], New York University Langone Hospital—Long Island [NYU-LI], Hôpital Paris-Saint Joseph [HPSJ], and Instituto de Infectologia Emílio Ribas [IIER]), grouped by type of procedure: capsule endoscopy (CE 407/2020), device-assisted enteroscopy (CE 188/2021), endoscopic ultrasound (São João CE 41/2021, PI 153/22, and S22-00910), direct single-operator cholangioscopy (CE 41/2021, PI 153/22), and high-resolution anoscopy (IRB 00012157 and SPTC 81/2023). A team with Data Protection Officer (DPO) certification (Maastricht University) confirmed the non-traceability of data and conformity with the general data protection regulation (GDPR).

#### 2.1.1. Capsule Endoscopy

For this study, we included images from three different capsule endoscopy (CE) devices: PillCam SB3™ (Medtronic, Minneapolis, MN, USA), PillCam Crohn’s Capsule (Medtronic, Minneapolis, MN, USA), and OMOM HD™ Capsule Endoscopy System (Jinshan, Yubei, Chongqing, China). We included CE procedures focusing on small bowel evaluation as well as panendoscopy exams. Two hundred sixty images, corresponding to 100 patients from a single center (SJUH, Porto, Portugal), were retrieved for submission with a standardized prompt on ChatGPT.

Capsule endoscopy images were divided according to anatomical landmarks, and four groups of images were created: esophagus, stomach, small bowel, and colon. The groups containing esophageal and gastric images were labeled as showing normal mucosa or pleomorphic lesions. The latter category included vascular lesions (angiectasia, varices, and red spots), protruding lesions, ulcers, and erosions. For the small bowel group, six categories were considered: normal mucosa, xanthelasma or lymphangiectasia, ulcers or erosions, protruding lesions, and blood or hematic residues. Finally, the images were labelled into three categories for the colonic segment: normal mucosa, blood or hematic residues, and colonic mucosal lesions. The latter category included ulcers, erosions, vascular lesions (red spots, angiectasia, and varices), and protruding lesions (e.g., polyps, epithelial tumors, submucosal tumors, nodes). Each included frame was reviewed by three experts in CE, each having read more than 500 exams previously (M.M.S., H.C., and P.A.). The final labelling of the frame according to each category required unanimous classification by the three experts. Frames for which a consensus could not be obtained were excluded from the analysis.

#### 2.1.2. Device-Assisted Enteroscopy

Images from two distinct device-assisted enteroscopy (DAE) systems were used in this study: Fujifilm EN-580T (Fujifilm Corp, Tokyo, Japan) and Olympus EVIS EXERA II SIF-Q180 (Olympus Corp, Tokyo, Japan). The procedures were performed by two endoscopists from a single center with expertise in this technique. Images from the stomach, small bowel, and colon were obtained. A total of 200 images from 72 DAE exams performed at a single center (SJUH, Porto, Portugal) were included. Each included frame was classified dichotomously as displaying normal mucosa or any visible lesion, which included ulcers or erosions and vascular and protruding lesions.

#### 2.1.3. Endoscopic Ultrasound

A total of 100 endoscopic ultrasound images (EUS) from 48 patients from three different centers (SJUH, Porto, Portugal; HUPH, Madrid, Spain; NYU-LI, New York, NY, USA) were selected for characterization by ChatGPT. The EUS procedures were performed using three different linear echoendoscopes: Olympus^®^ GF-UCT180 (Olympus Corp, Tokyo, Japan), Olympus^®^ GF-UC140 (Olympus Corp, Tokyo, Japan), and SonoScape^®^ EG-UC5T (Sonoscape Medical Corp, Shanghai, China). Two different ultrasound processors were used: Olympus^®^ EU-ME2 ultrasound processor and SonoScape^®^ S60 Ultrasound System.

The retrieved images were divided into two subsets: a subset of EUS images showing pancreatic cystic lesions (n = 60) and another with pancreatic solid lesions (n = 40). Each image was ultimately classified as showing a mucinous or non-mucinous cystic lesion for the group of cystic lesions. Pancreatic cystic lesions were considered mucinous if cytology revealed mucinous epithelial cells or, in their absence, CEA fluid levels >192 ng/mL and glucose levels < 50 mg/dL. Regarding solid lesions, each frame was labeled as displaying either pancreatic adenocarcinoma lesions (PDACs) or pancreatic neuroendocrine tumors (PNETs). The diagnosis of each entity required histopathological evidence, either from an EUS-guided biopsy or surgical specimen.

#### 2.1.4. Digital Single-Operator Cholangioscopy

ChatGPT was queried to assess digital single-operator cholangioscopy (DSOC) images and predict the presence of malignancy in biliary strictures. The DSOC procedures were performed to investigate indeterminate biliary strictures. A total of 120 images from 32 DSOC exams performed at three different centers (SJUH, Porto, Portugal; HUPH, Madrid, Spain; NYU-LI, New York, NY, USA) were included. All exams were performed using the Spyglass DS2™ system (Boston Scientific, Marlborough, MA, USA). The biliary stricture was classified as malignant if cytological or histological evidence (either from biopsy or surgical specimen) existed. A final diagnosis was obtained for benign strictures in the case of a negative histopathology result of biopsy or surgical specimens and no evidence of malignancy during a 6-month follow-up period after the procedure. Three subsets of images were designed for evaluation by the LLM. The first subset of images contained images showing either benign or malignant biliary strictures to assess the ability of ChatGPT to differentiate these two entities. Two additional subsets of images were created to assess the LLM’s performance in identifying morphological features associated with higher malignancy probability: “tumor vessels” and papillary projections. Each of the latter subsets contained images showing tumor vessels or papillary projections versus other findings of benign etiology.

#### 2.1.5. High-Resolution Anoscopy

The LLM was asked to evaluate high-resolution anoscopy (HRA) images and classify each image as demonstrating evidence of high-grade squamous intraepithelial lesion (HSIL) or low-grade squamous intraepithelial lesion (LSIL). The analysis was divided into four groups: unstained HRA images, staining with 5% acetic acid, staining with lugol, and after therapeutic manipulation of the anal canal. The latter subset of images included frames collected during in-office therapeutic procedures at different stages of completion, which were classified by experts as showing areas compatible residual lesions within areas of previously defined HSIL. The images were included from patients with histologically proven HSIL or LSIL, following the recommendations of the College of American Pathologists. Each group comprised 40 images representing the anal transformation zone, totaling 160 images from 32 patients from two centers (IIER, São Paulo, Brazil; HPSJ, Paris, France). The HRA exams were performed using two distinct HRA devices: a conventional colposcope (KLP 200 LED^®^, Kolplast, São Paulo, Brazil) and a high-resolution videoproctoscopy system (THD^®^ HRStation, THD SpA, Correggio, Italy).

### 2.2. Prompt Construction

We developed standardized prompts to instruct ChatGPT to provide predictions for each category. The construction of these prompts followed evidence on the best practices of prompt engineering [11]. It included elements for LLM instruction on the task to be performed, background context to shape the LLM’s responses, input data to be analyzed by the LLM, and indications on the expected output format. Prompts were adapted and inputted into the LLM by four gastroenterologists (T.R., F.M., M.M., and A.C.) who aggregated the outputs of the LLM. The output was provided in table format and comprised two columns (file name and prediction), which were later exported for comparison with the final diagnosis (true label). The prompts used in this study are shown in Table S1.

### 2.3. Statistical Analysis

ChatGPT 4’s prediction was compared to the definitive classification for each frame. The primary outcome was the overall diagnostic accuracy of the LLM for each query on capsule endoscopy, DAE, EUS, DSOC, and HRA. Secondary outcomes included sensitivity, specificity, and positive and negative predictive values. Additionally, for each exam type receiver operating curves (ROC) were developed, with determination of the area under the ROC curve for evaluating technology discriminatory ability. Statistical analyses were performed using SPSS Statistics v29.0 (IBM Corp., Armonk, NY, USA).

## 3. Results

### 3.1. General Description

Images from 5 technologies were selected for assessment by the LLM (n = 740). Regarding CE, a total of 260 images from 100 CE procedures using three different devices were collected (PillCam SB3™, n = 72; PillCam Crohn’s Capsule™, n = 23; OMOM HD™ Capsule, n = 5). These images were divided according to the anatomical regions, including images from the esophagus (n = 40), stomach (n = 40), small bowel (n = 120), and colon (n = 60). Regarding DAE, 200 images were included, originating from a pool of 72 DAE exams using two distinct systems: Fujifilm EN-580T (n = 49) and Olympus EVIS EXERA II SIF-Q180 (n = 23). For DSOC, 120 images from 32 exams were considered for this analysis. Concerning EUS, 100 images from 48 patients were submitted for assessment by ChatGPT. Finally, considering HRA, a total of 160 images from 32 patients were included in this study, arranged into four distinct groups: unstained HRA images (n = 40), after acetic acid staining (n = 40), after staining with lugol iodine (n = 40), and after therapeutic manipulation of the anal canal (n = 40).

#### 3.1.1. Capsule Endoscopy

Images from CE were divided into four anatomical regions: esophagus, stomach, small bowel, and colon.

##### Esophagus

The LLM model was required to identify and report the presence of any subtype of lesion. The overall diagnostic accuracy was 50.0%. The LLM reported all frames as showing a lesion, corresponding to a sensitivity of 100% and a specificity of 0% (AUC 0.50).

##### Stomach

Similarly to the esophagus, CE images showing gastric mucosa were fed into the LLM to assess its capability to identify the presence of gastric lesions. ChatGPT demonstrated a sensitivity, specificity, and overall accuracy of 90.0% for detecting gastric lesions in CE images (AUC 0.90).

##### Small Bowel

**Figure 1:**
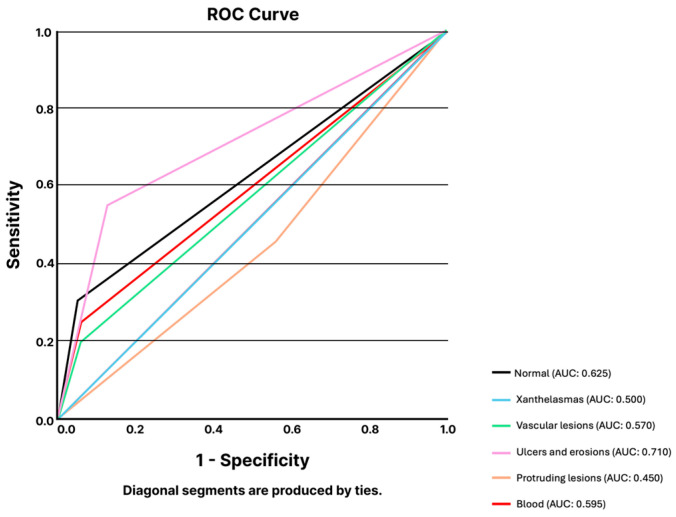
ROC analysis of ChatGPT-4’s performance for the classification of small bowel CE images across lesion categories. Abbreviations: AUC: area under the curve; CE: capsule endoscopy; ROC: receiver-operating characteristic curve analysis.

Regarding the small bowel, the LLM was asked to identify the most common types of small bowel lesions: xanthelasmas and lymphangiectasia, vascular lesions, ulcers and erosions, protruding lesions, and blood or hematic residues. The LLM showed a mean overall accuracy for the identification of small bowel lesions of 73.0% (95% CI 64.6–81.5%). The mean sensitivity and specificity were 64.9% (95% CI 49.6–80.2%) and 73.5% (95% CI 61.1–86.0%). The results are summarized in Table 1. The model had an overall AUC of 0.590, varying from 0.450 for identifying protruding lesions and 0.710 for detecting ulcers and erosions (Figure 1).

##### Colon

For colonic lesions, ChatGPT showed an overall accuracy of 52.8% (95% CI 0.487–0.569). The model showed similar performances for the detection of pleomorphic colonic lesions, with a sensitivity and specificity of 35.0% and 67.5%, respectively, and an overall accuracy of 56.7%. ROC analysis showed AUCs varying from 0.475 for identifying normal colonic mucosa to 0.513 for predicting the presence of pleomorphic lesions or hematic residues.

#### 3.1.2. Device-Assisted Enteroscopy

Images from DAE exams were shown to the LLM to predict the presence of any lesion. Images from several anatomical segments were included. From the 200 submitted images, the transformer model correctly labelled 134, corresponding to an overall accuracy of 67.0%. The AI model showed 74.0% and 60.0% sensitivity and specificity. The LLM had an AUC of 0.670 for differentiating between frames with and without lesions.

#### 3.1.3. Endoscopic Ultrasound

A total of 100 EUS images were included, 60 depicting mucinous lesions and the remaining showing solid lesions. For cystic lesions, 40 images showed non-mucinous lesions, while 20 had mucinous features. A similar number of images showing lesions with histologic evidence of PDAC (n = 20) or PNET (n = 20) was included for pancreatic solid lesions.

**Figure 2:**
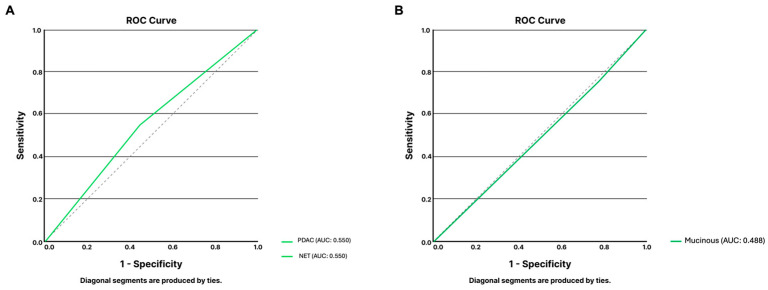
ROC analysis of ChatGPT-4’s performance for the classification of EUS images regarding solid lesions (**A**) and cystic lesions (**B**). Abbreviations: AUC: area under the curve; PDAC: pancreatic ductal adenocarcinoma; PNET: pancreatic neuroendocrine tumor; ROC: receiver-operating characteristic curve analysis.

Regarding mucinous lesions, the model demonstrated a sensitivity of 75%, a specificity of 22.5%, and an overall accuracy of 40.0% for identifying mucinous lesions and differentiating mucinous pancreatic cystic lesions. ROC analysis showed a poor discriminating capability of the algorithm for the main types of pancreatic cystic lesions (AUC 0.488, Figure 2A). This poor performance is related to, at least in part, the misclassification of images showing non-mucinous lesions for mucinous ones (31 out of 40 non-mucinous lesions).

Regarding solid lesions, the algorithm was asked to identify and differentiate EUS frames showing PDAC from those showing PNET lesions. Taking into consideration all included images (n = 40), this group was evenly divided into frames showing PDAC (n = 20) and PNET (n = 20) lesions. The LLM accurately classified 55.0% (n = 22) of frames. The model identified PDAC lesions with a sensitivity and specificity of 55.0%. The ROC analysis confirmed the limited performance of the model, with an area under the curve of 0.550 (Figure 2B).

#### 3.1.4. Digital Single-Operator Cholangioscopy

For DSOC, the aim was to assess the ChatGPT’s ability to differentiate between images showing malignant and benign biliary strictures as well as identify morphological features associated with biliary malignancy: papillary projections and tumoral vessels.

**Figure 3:**
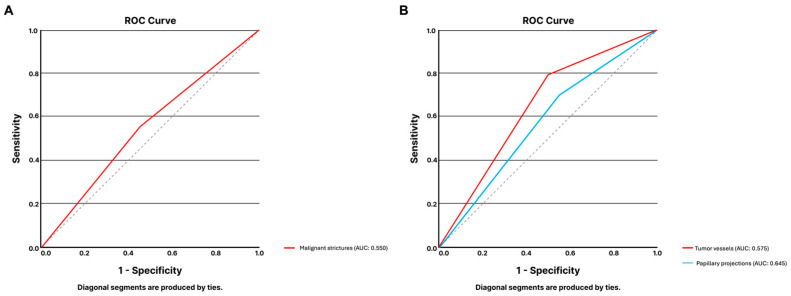
ROC analysis of ChatGPT-4’s performance for the classification of DSOC images regarding the presence of benign vs. malignant biliary strictures (**A**) and morphologic features associated with malignancy (**B**). Abbreviations: AUC: area under the curve; DSOC: digital single-operator cholangioscopy; ROC: receiver-operating characteristic curve analysis.

A total of 40 images showing biliary strictures were presented for analysis by the LLM, equally distributed between malignant and benign biliary strictures (each n = 20). The model had a sensitivity and specificity of 55.0% for detecting malignant biliary strictures. The system correctly labelled 55.0% of the frames. This limited performance was confirmed by the ROC analysis, with the model reaching an AUC of 0.550 (Figure 3A).

Two datasets were created to detect morphologic features associated with malignancy (papillary projections and tumoral vessels), with 39 and 40 frames, respectively.

Regarding papillary projections, the model correctly identified 25 of the 39 frames of that dataset (64.1%). The sensitivity and specificity of the model were 78.9% and 50.0%, respectively (AUC of 0.645, Figure 3B).

For the detection of tumoral vessels, from the 40 images comprising the dataset, 20 had evidence of malignant strictures portraying dilated tortuous vessels, and the remaining 20 frames referred to other findings of benign etiology. The LLM correctly identified 57.5% of frames. The algorithm achieved a sensitivity of 70.0% and a specificity of 45.0%. The AUC for the detection of tumoral vessels was 0.575 (Figure 3B).

#### 3.1.5. High-Resolution Anoscopy

**Figure 4:**
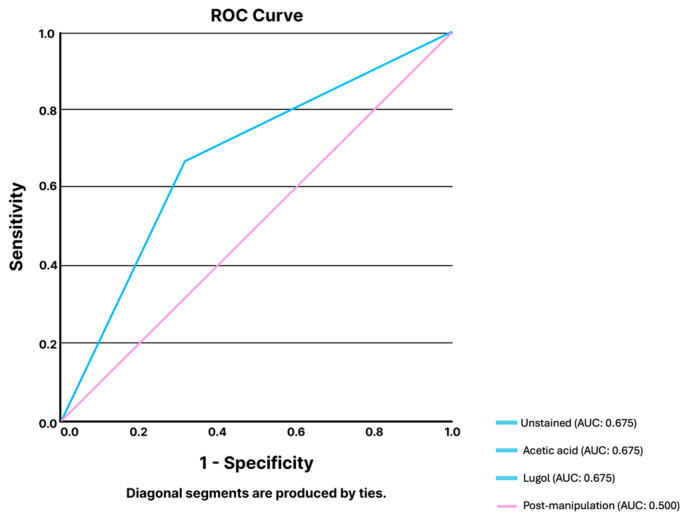
ROC analysis of ChatGPT-4’s performance for the classification of HRA images regarding the presence of HSIL vs. LSIL according to different staining stages and after therapeutic manipulation of the anal canal. Abbreviations: AUC: area under the curve; HRA: high resolution anoscopy; HSIL: high-grade squamous intraepithelial lesion; LSIL: low-grade squamous intraepithelial lesion; ROC: receiver-operating characteristic curve analysis.

A total of 160 HRA images were included. These images were divided according to different stages in the protocol for diagnosis and treatment of suspected anal squamous cell carcinoma precursors: unstained HRA images (n = 40), after 5% acetic acid staining (n = 40), lugol iodine staining (n = 40), and therapeutic procedures to the anal canal (n = 40). For each image, the LLM was required to categorize the frame as showing HSIL or LSIL. For unstained HRA images, the model correctly labelled 67.5% of frames. The model achieved a sensitivity of 66.7% and a specificity of 68.4% for identifying images showing HSIL. The AUC for identifying HSIL was 0.675 (Figure 4). From the 40 HRA images after acetic acid staining, the LLM correctly identified 11 of the 20 images showing HSIL, corresponding to a sensitivity of 52.4%. The specificity of the model was 52.6%. A total of 21 out of the 40 images were correctly labelled by the model, corresponding to an overall accuracy of 52.5%. The AUC for the identification of HSIL was 0.675 (Figure 4). From the total number of images with lugol iodine staining (n = 40), the LLM correctly labelled 19, corresponding to an overall accuracy of 47.5%. The model achieved a sensitivity of 95.0%. All images showing LSIL were erroneously classified as HSIL by ChatGPT (specificity 0%). The AUC for detecting HSIL in these procedural conditions was 0.675 (Figure 4). The group of HRA images immediately after interventions to the anal canal were used to identify residual HSIL lesions. From the 40 images, a total of 20 were correctly classified, corresponding to an overall accuracy of 50.0%. The model had a sensitivity and specificity of 50.0% for detecting HSIL, corresponding to an AUC of 0.500 (Figure 4).

## 4. Discussion

The evolution of AI systems has generated an enormous interest in medicine. The implementation of these algorithms, and particularly LLMs, aims to assist in several stages of healthcare, from medical education, triage, and clinical diagnosis to patient education [12]. In gastroenterology, most investigation has been dedicated to deep learning systems designed for detecting and characterizing lesions across distinct imaging methods, most importantly gastrointestinal endoscopy. Indeed, medical imaging plays a significant role in gastroenterology, and, therefore, most studies on deep learning algorithms in gastrointestinal endoscopy use neural architectures adapted for image analysis, most commonly CNNs [13]. The layout of these neural networks resembles the animal visual cortex by processing the information across multiple layers, which enables the extraction of numerous features. This explains the ability of CNNs to analyze spatial data such as images.

ChatGPT was developed by OpenAI (San Francisco, CA, USA) in 2022 as an LLM, whose primordial functionalities are understanding and generating human-like text. ChatGPT’s architecture is based on the Transformer Model, a neural network architecture specifically designed to handle sequential data such as text. Therefore, the ChatGPT primordial tool understands, generates, and interacts with textual inputs. In the latest ChatGPT-4 version, vision capabilities were introduced, allowing the software to perform multimodal analysis [14,15]. These functionalities are separate from the main LLM model and comprise image analysis and generation. While the latter functionality is possible by integrating generative AI models, the former is enabled by the integration of other deep learning algorithms, mainly CNNs.

ChatGPT is available as a user-friendly AI chatbot platform. Investigation with this tool has mainly evaluated the performance of the LLM instrument for clinical diagnosis. An exploratory approach using complex internal medicine clinical vignettes showed diagnostic accuracies for the definition of five differential diagnoses and the prediction of the definitive diagnosis with overall accuracies of 81% and 60%, respectively [16]. These accuracy values were comparable to those obtained by internal medicine specialists. For radiology cases, ChatGPT-4 achieved an overall accuracy of 49% in defining the three-item differential diagnosis list [2]. In the field of gastroenterology, these systems have mostly been assessed in the role of auxiliary to the diagnosis or for streamlining patient communication and evidence-based recommendations. In 2023, Henson and coworkers evaluated the ability of ChatGPT to provide adequate recommendations for patients with gastroesophageal reflux disease. The authors documented that the chatbot issued appropriate recommendations in more than 90% of cases [8]. More recently, Gorelik et al. created a customized GPT to provide evidence-based recommendations for the management of pancreatic cystic lesions, using a total of 60 clinical scenarios [17]. The customized algorithm issued adequate recommendations in 52 of the 60 clinical scenarios (86.7%), comparable to the rate of adequate recommendations provided by experts, with a high agreement rate between the LLM and gastroenterologists (Cohen’s kappa coefficients 0.61–0.65).

The introduction of image analysis features in the latest versions of ChatGPT has generated interest in assessing the performance of this tool for medical image analysis. Despite the interest, the results have been generally disappointing. For example, ChatGPT-4 has demonstrated suboptimal performance in interpreting chest CT scans, with an overall diagnostic accuracy of 56.8% [18]. Similar results were obtained for dermatoscopy images when ChatGPT-4 was required to differentiate between melanoma lesions and nonatypical benign nevi [19]. Despite these AI solutions’ widespread adoption and intuitive interface, significant concerns regarding their performance and consistency exist. This contrasts with the promising results provided by other AI architectures for medical image analysis, particularly CNNs, which are now being progressively adopted into clinical practice.

To our knowledge, this is the first study showcasing the performance of these systems for image analysis in gastroenterology. In this study, the authors explored the potential of the transformer model ChatGPT-4 for the automatic analysis of images from several diagnostic methods in gastroenterology. First, the LLM achieved highly variable results for CE, with overall accuracies ranging from 50.0% to 90.0%. Regarding the application to small bowel images, ChatGPT-4 achieved an overall mean accuracy of 73.0% for identifying five of the most common findings in capsule endoscopy. These results contrast with previous publications reporting the use of CNNs with this goal [20]. Our group assessed the performance of a denary CNN for identifying and differentiating small bowel lesions according to their bleeding potential [20]. The CNN achieved an overall accuracy of 99% and AUCs ranging between 0.97 and 1.00. Similar results have been previously published on a large retrospective series. While the latter study lacked the differentiation component, AI-assisted capsule endoscopy reading reached a sensitivity and specificity of 100% [21]. The results were similar to those of other endoscopic techniques in gastroenterology. For example, for EUS, ChatGPT-4 had an overall accuracy of 40.0% in differentiating images showing mucinous from non-mucinous pancreatic cystic lesions. This is in contrast with performance data from CNNs. In 2021, Vilas-Boas et al., using a dataset of 5505, achieved an accuracy of 98.5% in differentiating these types of lesions [22]. This prospect is similar for solid pancreatic lesions and other fields of pancreaticobiliary endoscopy. Indeed, for DSOC, significant evidence has been accumulating over the years on the excellent metrics of CNNs in distinguishing between malignant and benign biliary strictures, with accuracies up to 94.9% and AUCs ranging from 0.794 to 0.988 [23,24,25,26]. On the other hand, the LLM presents a poor accuracy in differentiating images showing different types of biliary strictures (AUC 0.550). Similar results were obtained when evaluating morphologic features suggestive of biliary malignancy. Finally, regarding non-endoscopic imaging techniques in gastroenterology, particularly HRA, similar results were obtained, with accuracies ranging from 47.5% to 67.5%, far from the results reported for CNNs across distinct staining protocols and HRA devices [27,28,29]. These suboptimal performance metrics must be considered before LLM clinical implementation for image analysis, as a misclassification could have a negative impact for the patient, either by missing a diagnosis (as in the case of malignant biliary stricture) or leading to unnecessary surgical or endoscopic interventions (namely when identifying a benign pancreatic lesion as malignant). Finally, a wrong prediction could lead to lack of trust in the LLM by both patient and doctors, reducing the potential clinical application of the technology [30,31]. Therefore, significant improvements must be achieved before LLM image analysis clinical implementation.

This study has some limitations. First, despite providing an innovative approach across several diagnostic techniques in gastroenterology, the total number of included images remained limited. Moreover, the analysis did not include some diagnostic techniques central to clinical practice in gastroenterology, specifically functional studies (e.g., high-resolution manometry). Subsequent studies should include these techniques. Additionally, this study focused only on the evaluation of a single LLM. Future studies will focus on the comparison between different LLM models, identifying the model with highest discriminatory ability for image analysis in gastroenterology. Finally, although a judicious approach for prompt engineering was applied during the study design, the authors are not able to rule out potential interferences introduced by the selected prompt in the expected results. In fact, LLM are known for having limitations in answering closed questions, specifically with when there is a need for detailed evidence [32,33]. The decision between specific diagnosis/image types could be challenging in the absence of previous specific training of the LLM, when compared to the more commonly applied deep learning models.

In conclusion, introducing commercially available LLMs enhanced with vision functions is expected to impact routine clinical practice. Our study critically evaluates ChatGPT-4’s ability to interpret medical images retrieved from diagnostic techniques in gastroenterology. Despite the recognizable results obtained for text analysis, the performance results obtained for image analysis remain suboptimal. Further refinement of these models is required before these models can be evaluated in a real-life clinical scenario.

## Figures and Tables

**Table 1:** ChatGPT-4’s performance for the detection of and differentiation between different categories of small bowel lesions.

	Sensitivity	Specificity	PPV	NPV	Accuracy
X/L vs. N, %	0.0	100.0	-	85.7	85.7
V vs. N, %	66.7	85.7	80.0	75.0	76.9
U/E vs. N, %	100.0	85.7	91.7	100.0	94.4
PR vs. N, %	81.8	40.0	50.0	75.0	57.7
Blood vs. N, %	100.0	66.7	62.5	100.0	78.6
V vs. X/L, %	100.0	0.0	57.1	-	57.1
U/E vs. X/L, %	100.0	-	100.0	-	100.0
PR vs. X/L, %	100.0	0.0	36.0	-	36.0
Blood vs. X/L, %	100.0	-	100.0	-	100.0
U/E vs. V, %	91.7	66.7	84.6	80.0	83.3
PR vs. V, %	90.0	28.6	47.4	80.0	54.2
Blood vs. V, %	100.0	66.7	71.4	100.0	81.8
PR vs. U/E, %	56.3	57.9	52.9	61.1	57.1
Blood vs. U/E, %	62.5	100.0	100.0	78.6	84.2
Blood vs. PR, %	41.7	90.0	83.3	42.9	51.9
N vs. All, %	30.0	95.0	54.5	87.2	84.2
X/L vs. All, %	0.0	100.0	-	83.3	83.3
V vs. All, %	20.0	94.0	40.0	85.5	81.7
U/E vs. All, %	55.0	87.0	48.5	90.6	81.7
PR vs. All, %	45.0	45.0	14.1	80.4	45.0
Blood vs. All, %	33.3	94.0	45.5	86.2	82.5

Abbreviations X/L—xanthomas or lymphangiectasia; PR—protruding lesions; U/E—ulcers or erosions; V—vascular lesions; PPV—positive predictive value; NPV—negative predictive value.

## Data Availability

The prompts used for Chat-GPT 4 analysis are presented in the Supplementary Files. Additional data are available upon reasonable request.

## Supplementary Materials

The following supporting information can be downloaded at: https://www.mdpi.com/article/10.3390/jcm14020572/s1, Figure S1. Flowchart of study design. Images from five distinct diagnostic techniques in gastroenterology (both endoscopic and non-endoscopic) were selected for analysis by ChatGPT-4. Each group of images was submitted for analysis along with a predetermined prompt (Supplementary Table S1). The output of ChatGPT-4 was later analyzed and compared with true labels. Abbreviations: HSIL: high-grade squamous intraepithelial lesion; LSIL: low-grade squamous intraepithelial lesion; PDAC: pancreatic ductal adenocarcinoma; PNET: pancreatic neuroendocrine tumor. Table S1. Prompts generated for submission in ChatGPT-4.
