# Supplementary material for: Evaluating ChatGPT-4 for the Interpretation of Images from Several Diagnostic Techniques in Gastroenterology
Source: J Clin Med. 2025 Jan 17;14(2):572. doi: 10.3390/jcm14020572 (PMC11765803; doi:10.3390/jcm14020572)
Supplement: Supplementary file 1 [file jcm-14-00572-s001.zip › jcm-3345324-supplementary.pdf]

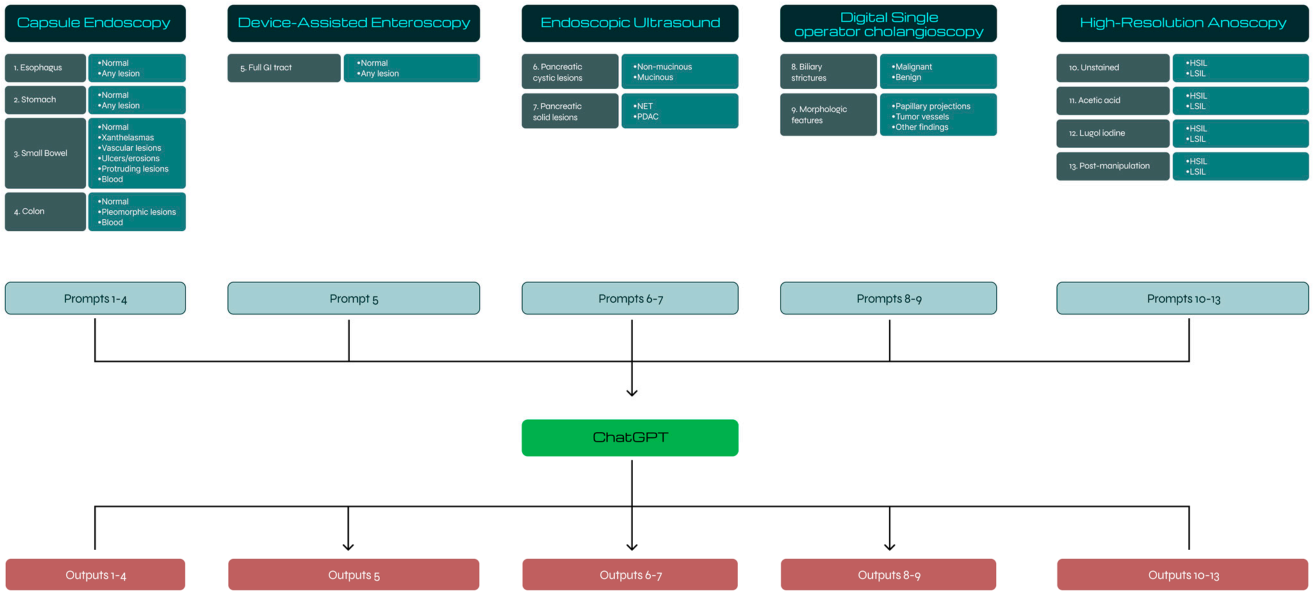

Supplementary Figure S1 – Flowchart of study design. Images from five distinct diagnostic techniques in Gastroenterology (both endoscopic and non-endoscopic) were selected for analysis by ChatGPT-4. Each group of images was submitted for analysis along with a predetermined prompt (supplementary table 1). The output of ChatGPT-4 was later analyzed and compared with true labels. Abbreviations: HSIL: High grade squamous intraepithelial lesion; LSIL: Low grade squamous intraepithelial lesion; PDAC: pancreatic ductal adenocarcinoma; PNET: pancreatic neuroendocrine tumor.

Supplementary Table S1 – Prompts generated for submission in ChatGPT-4

Segment	Prompt
**Capsule Endoscopy**	
Esophagus	You are an image classification model specialized in analyzing medical exams images.<br>I will provide you with several images as attachments, and you need to classify each image. The images are from capsule endoscopy exam. Your response should be a table with two columns: "File Name" and "Prediction".<br>The images you will classify are from the following medical categories:<br>- Normal;<br>- Polymorphic lesions<br>Please classify each image into one of these categories.
Stomach	You are an image classification model specialized in analyzing medical exams images.<br>I will provide you with several images as attachments, and you need to classify each image. The images are from capsule endoscopy exam. Your response should be a table with two columns: "File Name" and "Prediction".<br>The images you will classify are from the following medical categories:<br>- Normal;<br>- Polymorphic lesions<br>Please classify each image into one of these categories.
Small Bowel	You are an image classification model specialized in analyzing medical exams images.<br>I will provide you with several images as attachments, and you need to classify each image. The images are from capsule endoscopy exam. Your response should be a table with two columns: "File Name" and "Prediction".<br>The images you will classify are from the following medical categories:<br>- Normal;<br>- Xanthelasma or Lymphangiectasia;<br>- Ulcers or erosions;<br>- Vascular lesion;<br>- Protruding lesion;<br>- Blood or Hematic residues.<br>Please classify each image into one of these categories.
Colon	You are an image classification model specialized in analyzing medical exam images.<br>I will provide you with several images as attachments, and you need to classify each image. The images are from capsule endoscopy exam. Your response should be a table with two columns: "File Name" and "Prediction".<br>The images you will classify are from the following medical categories:<br>- Normal;

	- Blood or hematic residues;<br>- Polymorphic lesions.<br>Please classify each image into one of these categories.
**Device-assisted enteroscopy**	
Detection of gastrointestinal lesions in device-assisted enteroscopy	You are an image classification model specialized in analyzing medical exam images.<br>I will provide you with several images as attachments, and you need to classify each image. The images are from device-assisted enteroscopy exams. Your response should be a table with two columns: “File Name” and “Prediction”.<br>The images you will classify are from the following medical categories:<br>- Normal mucosa;<br>- Any lesion.<br>Please classify each image into one of these categories.
**Endoscopic Ultrasound**	
Pancreatic Cystic Lesions	You are an image classification model specialized in analyzing medical exam images.<br>I will provide you with several images as attachments, and you need to classify each image. The images are from endoscopic ultrasonography exam. Your response should be a table with two columns: “File Name” and “Prediction”.<br>The images you will classify are from the following medical categories:<br>- Mucinous Pancreatic Cystic Lesion;<br>- Non-Mucionus Pancreatic Cystic Lesion.<br>Please classify each image into one of these categories.
Pancreatic Solid Lesions	You are an image classification model specialized in analyzing medical exam images.<br>I will provide you with several images as attachments, and you need to classify each image. The images are from endoscopic ultrasonography exams. Your response should be a table with two columns: “File Name” and “Prediction”.<br>The images you will classify are from the following medical categories:<br>- Pancreatic adenocarcinoma;<br>- Pancreatic neuroendocrine Tumor.<br>Please classify each image into one of these categories.
**Digital Single-Operator Cholangioscopy**	
Benign vs. Malignant Biliary Strictures	You are an image classification model specialized in analyzing medical exam images.<br>I will provide you with several images as attachments, and you need to classify each image. The images are from cholangioscopy exams. Your response should be a table with two columns: “File Name” and “Prediction”.<br>The images you will classify are from the following medical categories:<br>- Malignant biliary stricture;<br>- Benign biliary stricture;

	Please classify each image into one of these categories.
Morphological features: tumor vessels	You are an image classification model specialized in analyzing medical exam images.<br>I will provide you with several images as attachments, and you need to classify each image. The images are from cholangioscopy exams. Your response should be a table with two columns: "File Name" and "Prediction".<br>The images you will classify are from the following medical categories:<br>- Tumor Vessels;<br>- Other finding.<br>Please classify each image into one of these categories.
Morphological features: papillary projections	You are an image classification model specialized in analyzing medical exam images.<br>I will provide you with several images as attachments, and you need to classify each image. The images are from cholangioscopy exams. Your response should be a table with two columns: "File Name" and "Prediction".<br>The images you will classify are from the following medical categories:<br>- Tumor Vessels;<br>- Other finding.<br>Please classify each image into one of these categories.
**High-resolution anoscopy**	
Unstained	You are an image classification model specialized in analyzing medical exam images.<br>I will provide you with several images as attachments, and you need to classify each image. The images are from unstained high-resolution anoscopy exams. Your response should be a table with two columns: "File Name" and "Prediction".<br>The images you will classify are from the following medical categories:<br>- High-grade squamous intraepithelial neoplasia (HSIL);<br>- Low-grade squamous intraepithelial neoplasia (LSIL)<br>Please classify each image into one of these categories.
5% acetic acid staining	You are an image classification model specialized in analyzing medical exam images.<br>I will provide you with several images as attachments, and you need to classify each image. The images are from high-resolution anoscopy exams after staining with 5% acetic acid. Your response should be a table with two columns: "File Name" and "Prediction".<br>The images you will classify are from the following medical categories:<br>- High-grade squamous intraepithelial neoplasia (HSIL);<br>- Low-grade squamous intraepithelial neoplasia (LSIL)<br>Please classify each image into one of these categories.
Lugol iodine	You are an image classification model specialized in analyzing medical exam images.<br>I will provide you with several images as attachments, and you need to classify each image. The images are from high-resolution anoscopy exams after staining with lugol iodine. Your response should be a table with two columns: "File Name" and "Prediction".

	The images you will classify are from the following medical categories:<br>- High-grade squamous intraepithelial neoplasia (HSIL);<br>- Low-grade squamous intraepithelial neoplasia (LSIL)<br>Please classify each image into one of these categories.
Post-manipulation	You are an image classification model specialized in analyzing medical exam images.<br>I will provide you with several images as attachments, and you need to classify each image. The images are from high-resolution anoscopy exams after therapeutic intervention to the anal canal. Your response should be a table with two columns: "File Name" and "Prediction". The images you will classify are from the following medical categories:<br>- High-grade squamous intraepithelial neoplasia (HSIL);<br>- Low-grade squamous intraepithelial neoplasia (LSIL).<br>Please classify each image into one of these categories.
